# Safety, tolerability and pharmacokinetic properties of intranasal artesunate in a first in human phase 1 trial

**DOI:** 10.1038/s41598-026-56855-1

**Published:** 2026-07-06

**Authors:** Karim Traore, Anne-Lise Bienvenu, Drissa Coulibaly, Abdoulaye Kassoum Kone, Amatigue Zeguime, Karim Bengaly, Mohamed Cisse, Joel Tarning, Mahamadou Ali Thera, Stephane Picot

**Affiliations:** 1 Parasites & Microbes Research & Training Center, Université des Sciences, Techniques et Technologies de Bamako, Bamako, Mali; 2 Université Lyon 1, ICBMS, UMR 5246 CNRS-INSA-CPE, Villeurbanne, France; 3 Service Pharmacie, Groupement Hospitalier Nord, Hospices Civils de Lyon, Lyon, France; 4 Centre for Tropical Medicine & Global Health, Nuffield Department of Medicine, University of Oxford, Oxford, UK; 5 Mahidol Oxford Tropical Medicine Research Unit, Faculty of Tropical Medicine, Mahidol University, Bangkok, Thailand; 6 Eunovis, Lyon, France; 7 Parasitology, Institut des Agents Infectieux, Hôpital de la Croix-Rousse, Hospices Civils de Lyon, Lyon, France

**Keywords:** Malaria, Artesunate, Nasal, Phase 1, Safety, Tolerability, Pharmacokinetic, Diseases, Drug discovery, Health care, Medical research

## Abstract

Malaria is responsible for more than half a million deaths each year. Parenteral artesunate is the recommended first-line treatment for severe malaria, but prompt treatment is often delayed affecting patient outcomes. Rectal artesunate is recommended as a pre-referral treatment but shows variable absorption and can be problematic when treating children with gastrointestinal disturbances. Nasal drug administration offers a non-invasive and user-friendly alternative that enables rapid drug absorption. A Phase I, first in human trial was conducted to evaluate the safety, tolerability and pharmacokinetic properties of increasing doses of a novel intranasal formulation of artesunate powder. Three dosing regimens were assessed in adult volunteers. Plasma samples were collected for up to 24 h post-dose for drug measurements of artesunate and dihydroartemisinin. Intranasal artesunate was safe and well tolerated. Only mild adverse events were reported shortly after drug administration. No severe adverse events were observed. Pharmacokinetic analysis confirmed rapid systemic absorption of artesunate but with high inter-individual variability. Dose-normalized exposures to dihydroartemisinin suggested a non-linear dose-exposure relationship. Further evaluation of this novel formulation of artesunate is needed to establish safety and efficacy in patients with malaria. This, first in human, phase 1 provides justification for subsequent phase 2 clinical trials.

**Supplementary Information:**

The online version contains supplementary material available at https://doi.org/10.1038/s41598-026-56855-1.

## Introduction

Malaria is one of the most important infectious diseases affecting humans. In 2024, it caused more than 500,000 deaths, most of which were young children in sub-Saharan Africa^1^. While most cases are uncomplicated and can be successfully treated with oral antimalarial drugs, these infections can develop into severe malaria in non-immune individuals, such as children under five years of age. Rapid and optimal antimalarial treatment is critical to improve the outcomes of severe malaria. Although considerable progress has been made in the fight against malaria over the past few decades, malaria-associated childhood mortality remains at an unacceptable level in endemic areas. Children with severe malaria commonly present with a history of a febrile illness lasting one to three days and with complications such as severe anemia, prostration, respiratory distress, shock, convulsions and impaired consciousness^2,3^. Children are hospitalized when critically ill with life-threatening complications, but access to intensive care is limited in many endemic areas, and the high volume of severe malaria cases poses a major challenge to resource-constrained hospitals^4^. Moreover, surveys report that 40% of febrile children do not even present for treatment at a clinic or hospital. Without treatment, severe malaria is frequently fatal, most often within the first 24 h after complications develop. For survivors, 5% to 30% experience long-term neurocognitive impairment. Severe malaria is a medical emergency that requires early diagnosis and treatment to prevent both death and neurologic disability^2^.

Parenteral (intravenous, IV; or intramuscular, IM) artesunate is currently the first-line therapy for severe malaria^2^. Parenteral administration is difficult to implement in peripheral health facilities, and even more so in small children. The parenteral route is invasive and requires aseptic technique, a supply of unexpired sterile catheters and tubing, and trained medical staff to ensure safe delivery and monitoring^5^. In this context, IV/IM administration of artesunate may be delayed or suboptimally implemented, leading to death and increased morbidity. Moreover, IV/IM drug administration requires qualified nurses or doctors who are often unavailable in many remote areas where malaria is common, leaving children who live in these areas at high risk for poor outcomes.

Rectal administration of artesunate is an alternative route already in use^6^. The World Health Organization (WHO) recommends rectal artesunate as pre-referral treatment before referring patients to an appropriate facility for parenteral treatment^7^. Although this route allows for systemic absorption, some individuals experience delayed diffusion into the circulation, leading to variable drug absorption and potential delayed antiparasitic activity^8,9^. The rectal route of administration is also complicated as many patients with malaria infections display gastrointestinal disturbances, resulting in unreliable absorption of the suppository. Rectal artesunate is indicated for children aged 6 months to under 6 years old. The findings from the phase 2 SEvere MAlaria with Rectal artesunate and Artemisinin Combination Therapy (SEMA-ReACT) project (https://mailchi.mp/mmv/sema-react-newsletter-september-10138079) reported that most children with severe malaria were treated at the community level with rectal artesunate and not referred to a health facility with available injectable artesunate. Furthermore, a shift in the age-specific susceptibility to malaria has been reported following population level interventions, such as effective treatments with artemisinin-based combined therapies (ACTs), long-lasting insecticide-treated nets (LLINs), rapid-diagnosis tests (RDTs), seasonal malaria chemoprevention (SMC), and intermittent preventive treatment in children (IPTc)^10,11^. After parenteral artesunate was implemented as first-line therapy for malaria, global mortality decreased significantly^12^. However, further gains in the reduction of mortality have been limited. Complementary solutions for early management of patients with severe malaria in Africa are urgently needed. To reduce case-fatality, effective treatment must be administered as soon as symptoms of severe malaria appear. For this to be possible, it is essential to have a simple and safe method of administering artesunate that allows rapid absorption of drug into the systemic circulation.

Nasal drug administration offers a noninvasive method that provides rapid drug absorption^13^. The high vascularisation and permeability of the nasal mucosae enable highly efficient systemic drug delivery via this route. The nasal cavity is supplied with blood by branches of the ophthalmic, sphenopalatine and facial arteries, and by extensive anastomoses in Kiesselbach’s plexus. Venous return drains via the superior vena cava into the right heart chambers, avoiding a hepatic first-pass effect. The pharmacokinetic properties of a drug administered via the intranasal route depend on nasal conditions, including mucociliary clearance, drug viscosity, and volume administered^14,15^. However, variations in inspiratory flow or rhinitis have only a minor influence on the efficacy of drug disposition^16^. Drug delivery via nasal route is limited by several factors, including a short drug retention time due to mucociliary clearance, metabolic degradation within the nasal cavity, and constraints on dosing volume^17^. Despite these limitations, over the last two decades, the nasal route has been successfully used to deliver diverse pharmaceutical products, including vaccines (influenza vaccine), hormones (insulin, melatonin, progesterone), analgesics (morphine), cardiovascular drugs (propranolol), and anti-inflammatory drugs (indomethacin)^18–20^. The U.S. Food and Drug Administration (FDA) approved intranasal sumatriptan powder for acute migraine treatment in adults in 2016^21^. Compared with oral administration, the intranasal route provides more rapid and superior relief of migraines symptoms^22^. Another widely adopted intranasal therapy is the opioid reversal drug naloxone, which is now widely available to lay persons who safely and effectively administer the drug to affected persons outside of the medical care setting, saving lives^23^. The FDA has recently approved an epinephrine nasal spray for treating severe allergic reactions. It has been successfully tested on young children weighing 15 kg^24^. No differences in the pharmacokinetic parameters were observed between children and adults for the recently approved epinephrine treatment when administered intranasally.

We previously tested the safety, pharmacokinetic and efficacy of intranasal administration of artesunate in experimental cerebral malaria^25^. Intranasal artesunate was not responsible for either local or general toxicity. Artesunate and dihydroartemisinin (DHA) reached a T_max_ and a C_max_ in less than 15 min in blood and DHA was detected in brain 15 min after administration. Furthermore, the parasitemia was decreased by 89% within the first 24 h after a single dose of nasal artesunate^25^.

Recently, we investigated the ability of artesunate powder to traverse nasal multilayer epithelial cells without causing local toxicity and reach the olfactory and respiratory zones of the nose when sprayed into the nostrils^26^. We demonstrated that the anhydrous powder formulation of artesunate exhibited superior permeation efficiency compared to the standard solution of this drug. The powder formulation prevents artesunate from chemical degradation and increases its stability. These results confirm the feasibility of intranasal artesunate delivery as a potential therapeutic strategy for severe malaria. A nasal powder formulation offers unique benefits, including greater product stability, enhanced bioavailability, and the capacity to efficiently deliver molecules with low aqueous solubility. The objectives of this study were to evaluate the safety, tolerability and pharmacokinetic properties of increasing doses of the investigational medicinal product (IMP) (0.5, 1.0 and 2.4 mg/kg) in three groups of four healthy adults, aged 18–25 years, in Bamako, Mali.

## Results

### Volunteers

**Fig. 1:**
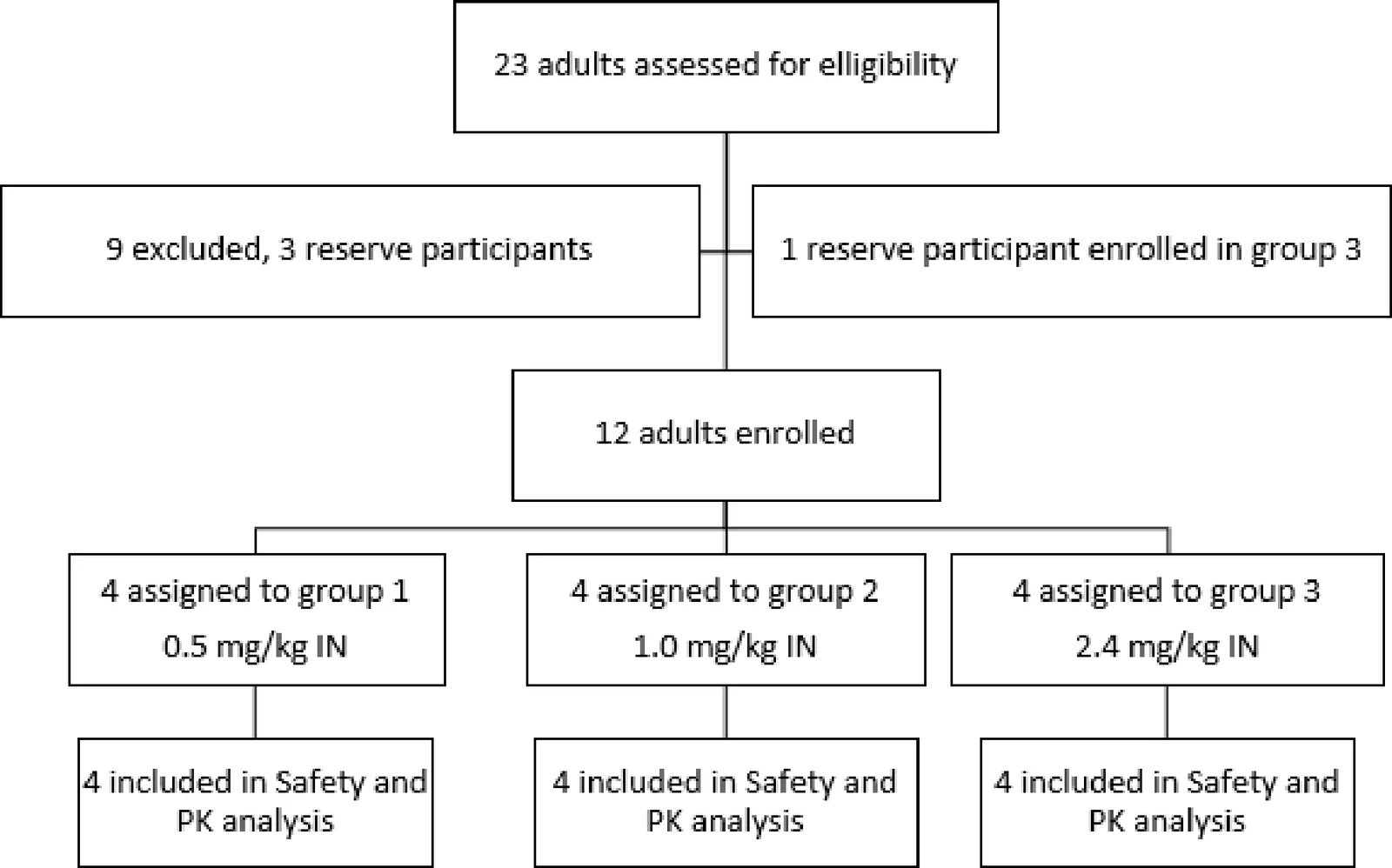
Study design and enrolment. A group of volunteers was assessed for eligibility few days before the administration of the first dose. Fifteen volunteers reached the inclusion criterias and were allocated to groups 1–3 doses according to their bodyweight. The full list of inclusion/exclusion criteria can be found in supplementary material. There was one protocol deviation among the volunteers from group 3.

Twenty-three participants were screened for eligibility in October 2024. Twelve adult volunteers were enrolled between October and November, 2024, and were sequentially assigned to group 1 (0.5 mg/kg), group 2 (1.0 mg/kg) and group 3 (2.4 mg/kg) (Fig. 1). Baseline characteristics were similar between the three study groups (Table 1). There were no obvious differences in age, temperature, weight, height, and body mass index among the three groups. One third of the participants were women, and all participants were Black African. The mean age was 21.8 ± 2.2 years (range 18–25), and the mean body mass index was 20.9 ± 2.9 kg/m^2^ (range 17–26).

**Table 1:** Baseline characteristics of included volunteers.

	Group 1 (*n* = 4) 0.5 mg/kg	Group 2 (*n* = 4) 1.0 mg/kg	Group 3 (*n* = 4) 2.4 mg/kg	Total (*n* = 12)
Women/men (%)	0/4 (0%)	1 / 3 (25%)	3 / 1 (75%)	4/8 (33%)
Mean age, years	20.5 (18–24)	21.5 (21–24)	22 (19–25)	21 (18–25)
Mean body temperature (°C)	36.2 (35.5–36.5)	36.1 (35.5–37)	36.1 (35.6–36.6)	36.1 (35.5–37)
Mean weight, kg	62.5 (53–85)	62.0 (54–65)	58.0 (46–77)	61.5 (46–85)
Mean height, m	1.7 (1.7–1.8)	1.7 (1.7–1.8)	1.7 (1.6–1.7)	1.7 (1.6–1.8)
Mean BMI, kg/m^2^	21.5 (18–26)	20.0 (19–22)	21.5 (18–27)	21 (18–27)

One-third of the participants were female and were mostly allocated to Group 3 due to the limited amount of artesunate that could be injected into the nostrils. Unfortunately, one of the female volunteers did not show up on the day of the trial, so she was replaced by the only other volunteer who was available in time. This reserve volunteer was a man whose body weight was higher than expected (77 kg).

No clinically relevant changes or statistical differences (*p* > 0.5) were seen in dynamic haematology parameters between screening (pre-dose), 8 and 24 h after the drug administration (Table 2).

**Table 2:** Hematology parameters of the volunteers at screening, 8 and 24 h after intranasal administration.

Parameters	Screening (mean ± SD)	8 h after (mean ± SD)	24 h after (mean ± SD)
0.5 mg/kg of artesunate (*n* = 4)			
WBC (x10^3^)	4.56 (0.94)	6.46 (0.74)	6.17 (0.39)
RBC (x10^6^)	5.18 (0.19)	4.95 (0.12)	5.16 (0.49)
Hemoglobin (g/dL)	13.85 (0.64)	12.12 (1.19)	12.55 (0.89)
Hematocrit (%)	45.07 (1.89)	45.15 (3.69)	47.42 (2.57)
MCV (fL)	86.92 (1.17)	90.85 (5.31)	91.90 (5.41)
Platelets (/mm^3^)	265.35 (22.79)	334.23 (41.24)	343.17(45.01)
Lymphocytes (/mm^3^)	1.88 (0.24)	2.45 (0.24)	2.32 (0.20)
Neutrophils (/mm^3^)	2.06 (0.68)	2.48 (0.25)	2.34 (0.26)
Basophils (/mm^3^)	0.02 (0.005)	0.06 (0.012)	0.045 (0.009)
Eosinophils (/mm^3^)	0.19 (0.041)	0.78 (0.29)	0.79 (0.31)
Monocytes (/mm^3^)	0.42 (0.09)	0.67(0.64)	0.65 (0.07)
1.0 mg/kg of artesunate (*n* = 4)			
WBC (×103)	4.88 (0.43)	7.39 (0.75)	6.51 (0.36)
RBC (×106)	5.27 (1.94)	4.91(0.09)	5.03 (0.13)
Hemoglobin (g/dL)	13.67 (0.87)	13.23 (0.64)	13.42 (0.80)
Hematocrit (%)	44.42 (2.40)	47.62 (2.39)	49.25 (2.78)
MCV (fL)	84.47 (4.58)	96.92 (3.95)	97.75 (3.62)
Platelets (/mm^3^)	297.17 (10.17)	276.05 (32.45)	287.85 (32.78)
Lymphocytes (/mm^3^)	2.12 (0.10)	2.93 (0.44)	2.38 (0.24)
Neutrophils (/mm^3^)	1.69 (2.67)	3.27 (0.41)	2.98 (0.32)
Basophils (/mm^3^)	0.02(0.004)	0.05 (0.009)	0.05 (0.010)
Eosinophils (/mm^3^)	0.46 (0.15)	0.52 (1.17)	0.55 (0.161)
Monocytes (/mm^3^)	0.60 (0.06)	0.60 (0.08)	0.54 (0.08)
2.4 mg/kg of artesunate (*n* = 4)			
WBC (x10^3^)	6.82 (1.34)	6.04 (0.70)	5.62 (0.80)
RBC (x10^6^)	5.53 (0.24)	4.87(0.28)	4.92 (0.23)
Hemoglobin (g/dL)	12.85 (1.09)	13.65 (0.90)	14.0 (0.87)
Hematocrit (%)	45.02 (5.06)	48.47 (3.56)	49.20 (3.52)
MCV (fL)	77.62 (4.58)	99.40 (3.63)	99.60 (3.54)
Platelets (/mm^3^)	323.90 (41.22)	359.95 (42.21)	362.07(48.89)
Lymphocytes (/mm^3^)	2.69 (0.38)	2.45 (0.44)	2.12 (0.40)
Neutrophils (/mm^3^)	3.0 (1.03)	2.80 (0.35)	2.72 (0.51)
Basophils (/mm^3^)	0.02(0.006)	0.06 (0.012)	0.05 (0.010)
Eosinophils (/mm^3^)	0.52 (0.36)	0.17 (0.009)	0.16 (0.009)
Monocytes (/mm^3^)	0.59 (0.07)	0.56 (0.10)	0.56 (0.11)

Biological analysis were performed first during the screening of volunteers, and then 8 h and 24 h after the intranasal administration of the IMP. There were no clinically meaningful effects on hematological parameters and no statistical difference between the three sampling times (student t-test). WBC, White blood cell count; RBC, Red blood cell count; MCV, Mean corpuscular volume.

### Safety and adverse events

**Fig. 2:**
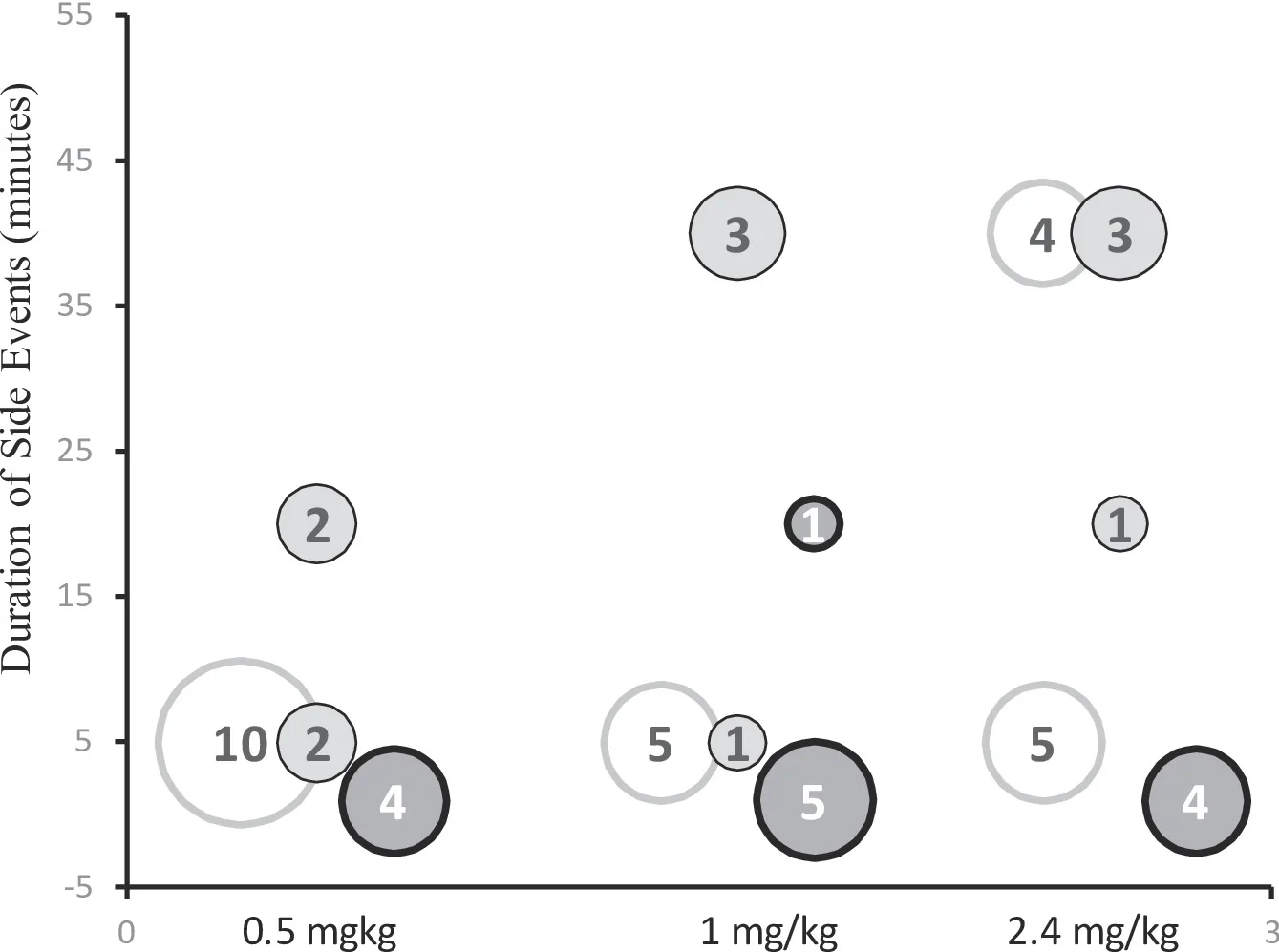
Distribution of the main adverse events in Nose, Throat and eyes by duration (minutes) and number of events (size of circles) according to the artesunate doses administered via the nasal route. Adverse events were classified as Nasal (white circles) for nasal irritation, nasal burning sensation and nasal congestion; Throat (light grey circles) for bitter taste and throat irritation; Eyes (dark grey circle) for ipsilateral or bilateral tearing. The figures inside circles are the number of events at each time.

No participant withdrew from the study. In the absence of a placebo group, which was considered difficult to implement, all treatment-emergent adverse events (TEAEs) were considered drug-related. All participants experienced at least one adverse event related to artesunate nasal administration, and a total of 50 adverse events were observed during the follow-up (Fig. 2). None of the adverse events were severe, and none required medical treatment. Almost all of the adverse events were classified as mild (94%), with only three (6%) classified as moderate (headache and ipsilateral tearing). The most frequent adverse events were a bitter taste in the throat (11/50) and a burning sensation in the nose (10/50). Ipsilateral (8/50) or bilateral (4/50) tearing and nasal discomfort (4/50) were also noted. The majority of adverse events occurred as a direct consequence of nasal administration, twenty-one events reported within five minutes (42%) and sixteen reported at five minutes (32%) post-administration. Only five adverse events persisted for up to 20 min (10%), and eight persisted for 40 min (16%) post-administration. The total number of adverse events did not increase with increased IMP doses administered and were 18 (36%), 15 (30%) and 17 (34%) in group 1, 2 and 3, respectively (Fig. 2). The cumulative duration of adverse events increased from 30 min post-administration for group 1 (7.5 min/ person), to 150 min (37.5 min/person) and 110 min (27.5 min/person) for group 2 and 3, respectively. There were no effect on hematological parameters measured 8 and 24 h after the IMP administration (Table 2), and no effect on QTc and heart rate. The risk of delayed hemolysis for non-infected volunteers was deemed irrelevant and hematological parameters were not measured at the end of the study visit at day 7.

### Pharmacokinetic study

The observed total assay coefficients of variation during bioanalytical drug measurements were below 6% for all of the quality control samples (low, medium, and high concentration), meeting FDA requirements of < 15%^27^. The pharmacokinetic properties of artesunate and DHA after nasal administration of artesunate are summarized in Table 3.

**Table 3:** Pharmacokinetic properties of artesunate and dihydroartemisinin, stratified by treatment arm, after intranasal administration of artesunate.

Parameter	Treatment arm		
	0.5 mg/kg (*n* = 4)	1.0 mg/kg (*n* = 4)	2.4 mg/kg (*n* = 4)
Artesunate			
C_max_ (ng/mL)	84 (58–192)	191 (168–370)	377 (208–558)
T_max_ (h)	0.08 (0.08–0.33)	0.08 (0.08–0.33)	0.08 (0–0.08)
AUC_last_ (h×ng/mL)	50 (30–68)	101 (69–155)	190 (116–286)
AUC_inf_ (h×ng/mL)	51 (31–69)	101 (70–155)	194 (117–287)
T_½_ (h)	0.77 (0.62–1.12)	0.49 (0.22–0.51)	0.9 (0.43–1.81)
Dihydroartemisinin			
C_max_ (ng/mL)	50 (41–62)	158 (91–196)	381 (139–439)
T_max_ (h)	0.84 (0.33–2)	0.5 (0.33–1)	0.84 (0.67–2)
AUC_last_ (h×ng/mL)	98 (71–112)	211 (148–428)	662 (340–889)
AUC_inf_ (h×ng/mL)	100 (71–113)	212 (149–431)	672 (401–913)
T_½_ (h)	0.65 (0.54–1.43)	0.75 (0.69–1.07)	1.32 (0.99–2.65)

C_max_ is the maximum observed plasma concentration; T_max_ is the time to maximum plasma concentration; AUC_last_ is the area under the drug concentration-time curve from time zero to the last measurable drug concentration; AUC_inf_ is the total area under the drug concentration-time curve from time zero to infinity; T_½_ is the terminal elimination half-life. All values are presented as median (range).

**Fig. 3:**
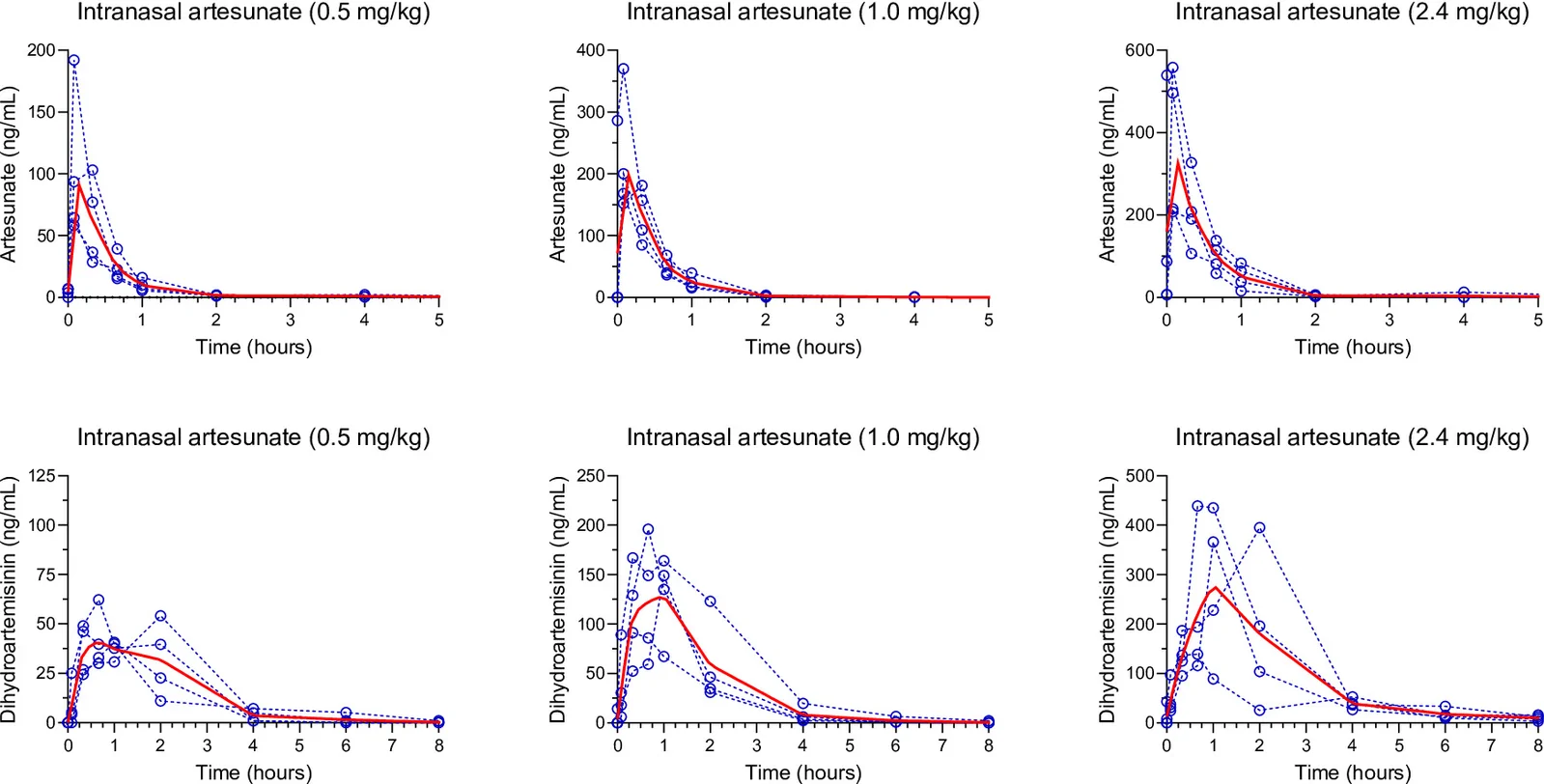
Concentration-time profiles of artesunate (upper panel) and dihydroartemisinin (lower panel) after intranasal administration of increasing doses of artesunate. (**A**–**C**) Pharmacokinetic profiles of artesunate after nasal administration of artesunate (0.5, 1.0, 2.4 mg/kg). (**D**–**F**) Pharmacokinetic profiles of dihydroartemisinin after nasal administration of artesunate (0.5, 1.0, 2.4 mg/kg). Dashed blue lines show individual concentration-time profile overlayed with a point-point spline average concentration-time profile (red solid lines).

**Fig. 4:**
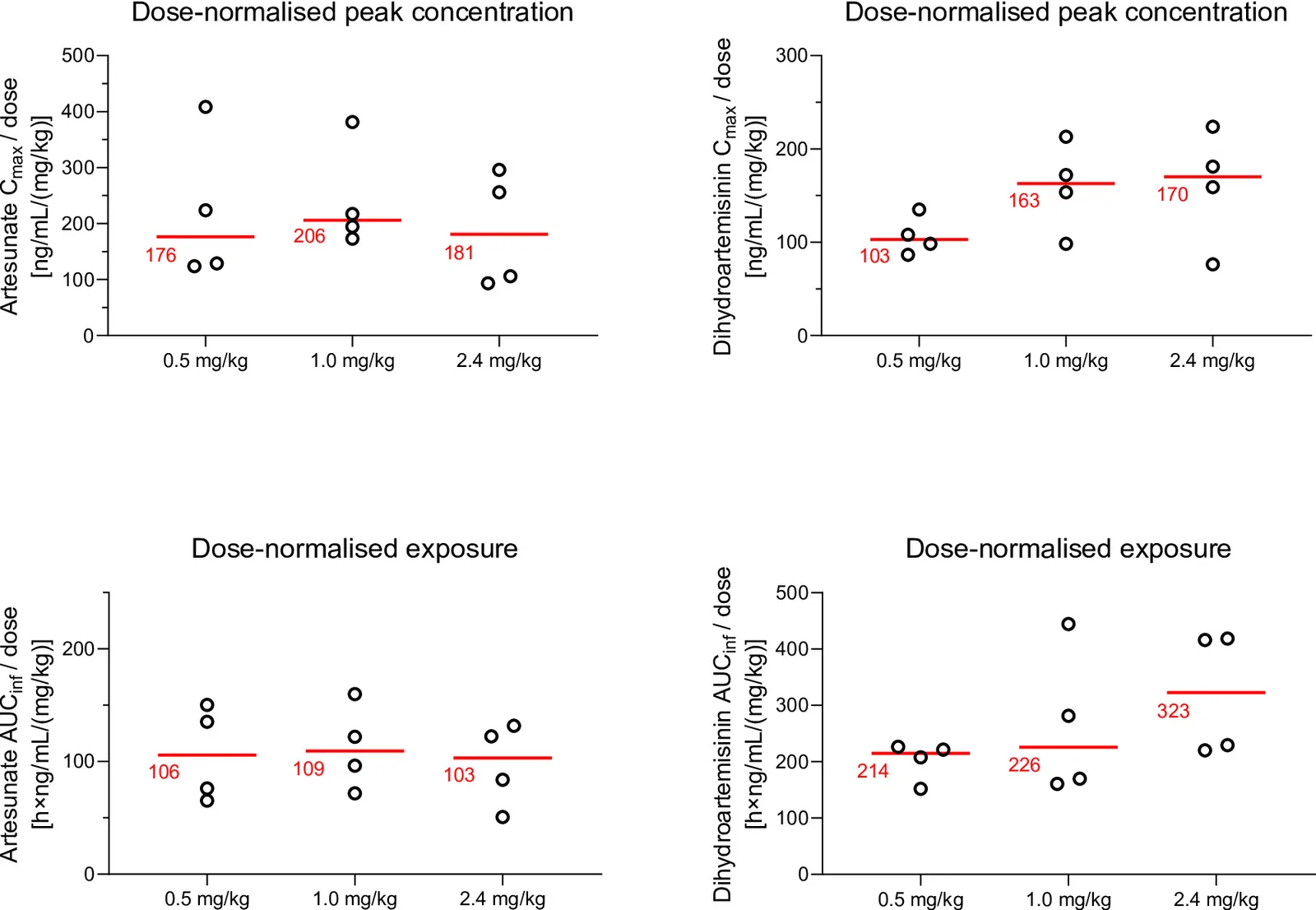
Dose-normalized peak concentration (upper panel) and exposure (lower panel) of artesunate (left panel) and dihydroartemisinin (right panel) after intranasal administration of increasing doses of artesunate. The peak concentration (**A**,**B**) and the total exposure (**C**,**D**) were normalised according to dose to account for the wide range of body weights among the volunteers and the underdosing of some of them. Circles indicate individually derived values, overlaid with the median value of the group. The median value is also typed in red above each group of data.

Briefly, artesunate was rapidly absorbed after nasal administration, with plasma concentrations peaking within 5 min of drug administration in almost all individuals (Fig. 3). Dihydroartemisinin is the major active metabolite of artesunate, peaking at around 40–60 min after intranasal drug administration of artesunate (Fig. 3). Concentration-time profiles showed considerable inter-individual variability and a trend towards non-linear pharmacokinetics, with higher dose-normalized exposure and peak concentration to DHA with increasing doses (Fig. 4). There was also an accompanying increase in the terminal elimination half-life of DHA with increasing doses. This was not seen with artesunate, suggesting that the absorption of artesunate remained relatively constant with increasing doses. However, the small number of participants and the variability preclude a definitive conclusion. The increase in the half-life of artesunate compared to DHA in group 1 is likely to be irrelevant and is probably due to delays in taking blood samples from the first four volunteers to be included in the study.

One participant enrolled in group 3 (2.4 mg/kg) did not present for IMP administration on the day of drug administration, and was replaced by the first reserve volunteer available. The weight of this reserve participant was 77 kg, requiring administration of 180 mg of artesunate to reach a weight-normalized dose of 2.4 mg/kg. This was not possible, and the volunteer received the maximum total dose of 140 mg (70 mg per nostril), resulting in a dose of 1.81 mg/kg (25% underdosing), which was considered as a minor protocol violation. This participant could not be reassigned to another group because the inclusion in lower-dose groups had already been completed. However, the values of his first artesunate and DHA plasma concentrations were higher than expected compared to other participants due to an interval of a few minutes between administrations in each nostril, responsible for a delay in the first blood sampling.

### Accelerated stability studies

In line with the International Council for Harmonisation of Technical Requirements for Registration of Pharmaceuticals for Human Use (ICH), an accelerated stability assessment program (ASAP) was conducted to evaluate the IMP shelf life under thermal and humidity conditions frequently encountered in endemic areas. Eighteen months after batch preparation, approximately 97% of the artesunate remained stable, and 3% was transformed to DHA in the three batches under the accelerated stability assessment program. These data confirm the remarkable stability of artesunate powder samples loaded in nasal devices, at 25 °C ± 2 °C / 60% relative humidity ± 5% and 40 °C ± 2 °C / 75% relative humidity ± 5%, at concentrations ranging from 20 to 70 mg per device.

### Assessing volunteer perceptions of anti-malaria treatment

One week after nasal administration, we conducted face-to-face structured interviews to assess participants’ perceptions of anti-malaria treatment via the nasal route. Four of the 12 participants had not previously experienced malaria. Five participants (41.6%) had previously received intravenous treatment and three (25%) had received oral medication. All participants considered the current malaria treatment administration routes to be tiring, whether intravenous or oral. Only two participants (16.6%) had previously received nasal treatment for another disease and found it uncomfortable. Of the eight participants who had been previously treated for malaria, 100% preferred the nasal route because it was easy (7), fast (3), and avoids injections (3).

## Discussion

This is the first report on the safety and pharmacokinetics of intranasally administered artesunate dry powder in humans. This study corroborates preclinical study findings in experimental severe malaria, and data obtained with in vitro culture of human nasal mucosa^25,26^. These preclinical studies showed that intranasal artesunate had no local or general toxicity in mice, a favorable pharmacokinetic and a high efficacy to reduce the parasite load by a factor of ten in few hours^25^. Artesunate permeation assays using transepithelial electrical resistance (TEER) through human nasal mucosa in vitro demonstrated that the powder formulation exhibited greater permeation efficiency than the solution^26,28^. Furthermore, spraying the artesunate powder into the artificial nostril on a nasal cast demonstrated that a significant amount of the drug reached the olfactory and respiratory zones^26^.

Using a dry powder increases the drug’s retention in the nasal cavity, thereby maximizing its contact with the mucosa, enhancing the penetration efficiency and diffusion as previously demonstrated^26^. Our study demonstrated that artesunate dry powder has a favorable thermal stability with an acceptable stability profile at 40 °C in high humidity conditions, compared to 25 °C for IV and rectocaps. Crystalline artesunate is thermally robust and is hydrolyzed to DHA under acidic conditions, with a half-life of 26 min at pH = 1.2 and 10 h at pH = 7.4^29,30^. The physiological nasal pH in humans is approximately 5.9 (4.9 to 7.4)^31^, resulting in a slow ex-vivo hydrolysis of artesunate to DHA. This makes the dry powder formulation better suited for tropical endemic conditions and easier to store^32^. Thus, it could reduce the time to effective treatment initiation which could improve outcomes in severe malaria patients.

The results from this first-in-human study demonstrate that a single 140 mg dose of artesunate powder can be administered intranasally (70 mg per nostril) without a safety signal. Treatment emergent adverse events (TAEEs) were frequent, but were predominantly mild and transient (lasting less than one hour). Adverse events were also reported following the intravenous, intramuscular and rectal administration of artesunate and these are considered to present an acceptable harm profile. The study demonstrated the feasibility of nasal administration of artesunate dry powder and indicated favorable participant acceptability. However, nasal irritation and a bitter aftertaste negatively affected overall acceptance, as revealed by the analysis of volunteer experience. Intravenous artesunate treatment has also been shown to cause a bitter taste in the mouth^33^, and is intended for patients experiencing severe symptoms, including coma. In such cases, the impact of local adverse events such as nasal irritation and a bitter taste may be less important. Nasal burning and bitter taste are also frequently observed with other drugs such as nirmatrelvir^34^ combined with ritonavir to treat SARS-CoV-2 infection, dihydroergotamine powder and other intranasal migraine products^35^.

The children’s perception of the intranasal route was assessed during treatment for other diseases. The acceptability of intranasal treatment among the paediatric population with type 1 diabetes was investigated in a comparison with injectable glucagon^35^. This study demonstrated that the intranasal treatment was easier to use, faster, safer, and more likely to succeed than the injectable treatment. A meta-analysis of intranasal analgesia for pain in children confirmed that children and parents prefer the intranasal route instead of intramuscular^36^. The acceptability of nasal route in malaria endemic area (The Gambia) was tested for influenza vaccination in children > 5 and was viewed favorably^37^. Moreover, a large cross-sectional study of different routes of administration according to population culture and beliefs, showed that the nasal route as a much acceptability than the rectal route^38^. The rectal route was chosen by 1% of participants in African-Islamic culture and was perceived to be the most painful or uncomfortable^38^. It is reasonable to conclude that children are likely to tolerate the administration of nasal artesunate in cases of severe malaria with impaired consciousness.

The pharmacokinetic profile of nasal artesunate powder is consistent with results obtained in studies of other nasal treatments, characterized by rapid absorption within minutes^39^. The results showed a rapid initial rise in plasma concentrations of artesunate and DHA due to the absorption of dry powder particles in the nasal cavities. A previously published study confirmed that the bioavailability of a monodispersed suspended drug particles did not differ for different insertion angles of 30, 40 or 50 degrees^40^, reducing the risk of altered drug deposition in the event of improper device positioning. This rapid absorption, resulting in high blood concentrations of artesunate and DHA within minutes is crucial for the pre-referral treatment of severe malaria as the drug rapidly needs to reach parasitized red blood cells to kill parasites and resolve symptoms. After rectal administration, it took a median (IQR) 30 min (15–45) for artesunate and 60 min (45–120) for DHA to reach peak plasma concentrations when administered to 83 children with severe malaria in the Democratic Republic of Congo^8^. The pharmacokinetic results showed a high between-subject variability, which is also seen in oral, parenteral and rectal dosing of artesunate from studies including numerous patients^8–42^. Concentration-time profiles showed a trend towards non-linear pharmacokinetics, with higher dose-normalized exposure and peak concentrations to DHA with increasing doses. There was also an accompanying increase in terminal elimination half-life of DHA with increasing doses. However, these trends were not seen with artesunate, suggesting that this is either a chance finding considering the small number of subjects and inter-individual variability or a result of saturated metabolic elimination of DHA. Moreover, the half-life of artesunate for the group of participants taking 0.5 mg/kg increased which is likely to be irrelevant and is due to delays in taking blood samples from the first four volunteers. Further studies in a larger patient cohort are needed to evaluate potential nonlinearities at higher doses. However, at this stage, these hypothesis are clearly exploratory, given that there are only four volunteers per group.

The pharmacokinetic parameters of artesunate after oral, rectal and intravenous dosing have been extensively studied^43–48^. A systematic review of artesunate and DHA pharmacokinetics compared intravenous, oral, rectal and intramuscular administration and reported that intravenous artesunate is associated with the highest initial artesunate and DHA concentrations (C_max_ = 3,260–29,677 ng/ml and 605–3210 ng/ml, respectively) and a half-life of 1.5 to 15 min for artesunate and 18–78 min for DHA^44^. These values were obtained following IV artesunate doses ranging from 0.5 to 8.0 mg/kg. After oral artesunate at standard doses of 4 mg/kg, C_max_ median data were 63–1040 ng/mL and 519–1850 ng/mL for artesunate and DHA, respectively. Following rectal artesunate of 10 mg/kg, C_max_ median data were 507–561 ng/mL and 682–1535 ng/mL for artesunate and DHA, respectively. Intranasal administration of 2.4 mg/kg resulted in median (range) peak concentrations of 377 (208–558) ng/mL and 381 (139–439) ng/mL for artesunate and DHA, respectively, thus clearly showing the ability to reach therapeutic concentrations (Table 4). The in vitro inhibitory concentration (IC50) of DHA was estimated to be between 0.1 and 2.0 µg/L^49^. After nasal administration of 2.4 mg/kg of artesunate, the C_max_ of DHA was higher than this threshold, reaching 380 µg/L (1341 nM). The AUC_inf_ of DHA was 672 h.µg/L (2363 nM). Although these values are lower than those obtained following intravenous administration of artesunate, they will likely lead to a significantly decrease in parasite burden in patients. Further testing of this hypothesis is required.

**Table 4:** Pharmacokinetic parameters of intranasal administration, compared to intravenous, oral, rectal and intramuscular administration data from previous systematic review^44^.

PK parameters	Administration route				
	Nasal (2.4 mg/kg)	IV (1.2–4 mg/kg)	PO (2–4 mg/kg)	IR (10–20 mg/kg)	IM (1.2–4 mg/kg)
	Mean (range)	Range	Range	Range	Range
C_max_ (ng/ml)					
ART	377 *(208–558)*	3260–29,677	62.9–1040	507–561	615–2195
DHA	381 *(139–439)*	605–3210	519–1850	682–1535	341–1166
T_max_ (h)					
ART	0.08 *(0–0.08)*	NA	0.33–1.5	0.8–1	7.2–12
DHA	0.84 *(0.33–2.0)*	0.008–0.2	0.5–2.04	1.5–2	25.9–45
t_1/2_ (h)					
ART	0.90 *(0.43–1.81)*	0.025–0.2	0.24–0.7	NA	25.2–48.2
DHA	1.32 *(0.99–2.65)*	0.3–1.31	0.64–4.02	0.79–1.8	31.9–52.7
AUC_0−∞_ (h×ng/ml)					
ART	194 *(117–287)*	49–1042	62.9–217	692–1076	535–856
DHA	672 *(401–913)*	418–2450	1160–2689	2403–5633	396–1496

ART, artesunate; DHA, dihydroartemisinin; PK, pharmacokinetic; CV, coefficient of variation; IV, intravenous; PO, per os; IR, intrarectal; IM, intramuscular; C max, maximal plasmatic concentration; T max, time to maximal plasmatic concentration; t1/2, half-life; AUC, area under the concentration-time curve; Dose-independent PK parameters are provided as range (CV) and dose-dependent parameters as range with the corresponding ART dose range (in mg/kg).

The earlier artesunate is administered, the greater its lifesaving benefit^50^. The route of administration does not affect its pharmacodynamic properties directly. The very first dose of artesunate is the most crucial, irrespective of the route of administration. Therapeutic concentration of artesunate and DHA after a single dose eliminate more than 99.9% of the parasite biomass within 24 h, and an even greater reduction is achieved within one asexual parasite cycle of 48 h. Although DHA concentrations exhibited high variability, the pharmacokinetic profiles closely resembled those observed following a standard oral dose of DHA-piperaquine which has demonstrated efficacy in the treatment of uncomplicated malaria^51^. The peak concentration (1341 nM) and the total exposition to DHA (2366 nM) after one intranasal treatment were in the same range than after rectal artesunate^52^. Selection of resistant parasites after a single nasal dose will provide very little resistance selective pressure, as demonstrated for rectal artesunate^50^. Due to its advantages, there may be a risk of misuse, which will require information for parents and training for village health workers. The next steps in the clinical development of intranasal artesunate for pre-referral treatment of severe malaria include evaluation of the safety, pharmacokinetics and parasitological efficacy of a single intranasal dose of artesunate in children with uncomplicated and severe malaria.

This study has some limitations. It was a single-center study with a limited number of adult participants and a short follow-up period which precludes definitive conclusions. Other limitations include the volunteer bias by recruiting adults living in the urban area of Bamako with a limited experience of malaria. Our study might also have been underpowered to detect adverse events and a placebo group is lacking. Despite these limitations, important safety and pharmacokinetic data was generated that support further clinical development.

In conclusion, this study highlights the potential for an innovative approach for pre-referral treatment of severe malaria in endemic areas, where options for rapid artesunate administration to children six years of age and older do not exist. Because intravenous access requires skilled medical personnel and sterile equipment, the standard treatment for severe malaria with intravenous artesunate is often delayed, and is frequently late or never received. Emergency administration of a lifesaving drug in a ready-to-use format, designed for use by non-medical personnel with minimal training, aims to overcome delays associated with referral and transfer to a health facility where injectable artesunate is available.

Intranasal artesunate spraying is proposed for emergency treatment of children with severe malaria, when intravenous or intramuscular AS is not immediately available and intrarectal AS cannot be used. Early treatment of severe malaria within minutes after symptom onset with one single dose of intranasal artesunate is expected to rapidly reduce the parasite load, prevent further parasite sequestration in the deep vasculature, and decrease risk of mortality and morbidity associated with severe malaria, including impaired neurocognitive development.

## Methods

### Study design and participants

This phase 1, first-in-human (FIH) trial was conducted at the Parasites and Microbes Research and Training Center, formerly known as Malaria Research and Training Center (Bamako, Mali). It was an open label, sequential ascending dose study. To enhance safety, the number of participants per group was limited to four as an empirical risk mitigation strategy^53^. While the majority of FIH clinical trials are placebo-controlled, the intranasal route of administration is very well tolerated for various drugs and the risk-to-benefit ratio of a placebo group was unclear.

Although artesunate has been widely used in humans, including at doses of up to 8 mg/kg, its safety as a powder has only been documented in experimental malaria in mice and in vitro using human nasal mucosa. Therefore, to mitigate the risk of toxicity, it was decided to start with a dose of 0.5 mg/kg, which is approximately one-fifth of the target dose. This target dose was 2.4 mg/kg, as recommended by WHO to treat severe malaria. This dose range was not broad enough to establish a no observed adverse effect level with a sufficient margin over the maximum exposure, but the safety of artesunate high doses was widely documented after intravenous, intramuscular, oral or rectal administration^44^.

Participants were healthy adult volunteers (males or non-pregnant, non-lactating females) aged 18–25 years (inclusive) from the Bamako area. None of them have a history of substance abuse or smoking. A specific contraception was not requested for women because artesunate is safe during pregnancy, rapidly eliminate and volunteers received only one single dose. Participants had to be able to comply with the study procedures and be in good health to be eligible for inclusion. Volunteers were screened for eligibility the days before study start, including assessments for serious acute or chronic illnesses using screening laboratory tests and a complete medical history. A trained physician performed a physical and nasal examination to check for nasal erythema, epistaxis, and septal perforation. Potential participants were excluded if they were immunosuppressed or had a history of recent malaria. The complete list of inclusion and exclusion criteria is presented in supplementary material.

The study was conducted in accordance with the Declaration of Helsinki, Good Clinical Practice (GMP), and in accordance with the law n°09-059 of December 28th, 2009, regulating biomedical research on human in Mali. Ethical clearance was obtained from the Ethics comittee of Université des Sciences, des Techniques et des Technologies de Bamako (USTTB), Faculty of medicine and odonto-stomatology, Point G Bamako 1805 Mali (Approval date: 24/04/2024). Written informed consent was provided by all participants before enrollment in accordance with the requirements of ICH E6 GCP. All information was disclosed in lay terminology and language that facilitates understanding of participants. The study is registered with Pan-African Clinical Trial Registry (Trial ID: PACTR202408719575384 (MALANOSE), Registration date: 01 Aug 2024).

### Study procedures

Twelve eligible participants were enrolled and sequentially allocated to groups 1–3. Study product was administered based on bodyweight (mg/kg). Three dosing regimens were assessed in three groups of four adults: 0.5, 1.0, and 2.4 mg/kg of artesunate as a single intranasal dose. A safety monitoring committee conducted safety oversight, including reviews of adverse event data from the first group of four participants receiving 0.5 mg/kg before dose escalation, and then again in participants receiving 1.0 mg/kg to ensure that no stopping rules were met before dose escalation to the subsequent dose group.

The IMP is a pure artesunate powder, purchased from WHO prequalified IPCA Laboratories (Mumbai, India) and provided by the Active Pharmaceutical Ingredient (API) supplier Hepartex (Saint-Cloud, France). Aptar Pharma’s Unidose (UDS) Nasal Powder system was used to deliver a single, accurate dose quickly and easily. UDS were filled with artesunate powder according to GMP by the Contract Development and Manufacturing Organization (CDMO) Skyepharma (Saint-Quentin-Fallavier, France). Doses ranged from 10 to 70 mg, and the devices were supplied ready-to-use at the study site. Each device was manually actuated with a fixed insertion depth of 1.5 cm, delivery angle (horizontal plane) of 45° and angle from the center wall of 0 to 5°. For doses exceeding 70 mg, the total dose was split into two devices, one for each nostril, and administered sequentially. Thus, the maximum dose available for this trial was 70 mg per nostril, resulting in a maximum total dose of 140 mg per person. Dosing regimens were stratified by body weight and study group. Participants weighing 50–59 kg received 25 mg in group 1 (0.5 mg/kg), 50 mg in group 2 (1.0 mg/kg) and 120 mg in group 3 (2.4 mg/kg). Participants weighing 60–69 kg received 30, 60 or 140 mg, respectively. Participants weighing 70–80 kg were assigned to groups 1 or 2, receiving 35 or 70 mg.

After study product administration, all participants were observed in the clinic for eight hours, with additional follow-up assessments at 24 h and on Day 7. These visits facilitated documentation of local and systemic safety parameters and pharmacokinetic sampling. Blood samples for chemical and haematological testing were immediately processed at the PMRTC CAP-certified Clinical Biology laboratory.

A 20G peripheral venous catheter (PVC) was placed to facilitate blood sampling and to reduce pain and discomfort for the first eight hours of the pharmacokinetic study. Blood samples (1 mL each) were collected immediately after drug administration, and at 5, 20, 40 min, and 1, 2, 4, 6, 8 h via the PVC, and at 24 h by direct venipuncture, yielding a total of 10 blood samples per volunteer. After each collection, the PVC was flushed with 2 mL saline, followed by a 20-second dwell period and vein occlusion for 15 s. A 1.0-mL syringe was then attached to withdraw 1.0 mL of blood. All samples were collected in tubes containing sodium fluoride/potassium oxalate anticoagulant. Tubes were pre-chilled before sampling, centrifuged at 4 °C, and stored immediately at − 80 °C. Samples were subsequently shipped on dry ice to the Mahidol Oxford Tropical Medicine Research Unit at Bangkok for artesunate/dihydroartemisinin drug quantification using a validated LC-MS/MS method^54^. The lower limits of quantification (LLOQ) were set to 1.19 and 1.96 ng/ml for ARS and DHA, respectively.

### Outcomes

The primary outcomes were the frequency, duration and severity of adverse events after the intranasal administration in the three dose groups. Adverse events were classified as mild (events that required minimal or no treatment and did not interfere with the participants’ daily activities); moderate (events that caused minor discomfort or concern); severe (events that hindered participants’ ability to perform their activities and might require systemic medication). A qualified medical doctor performed participant safety assessments, including vital signs measurement and a physical examination with special attention to the nasal cavities, the nasal mucosa, turbinates and nasal floor, and to identify any epithelial disruption or local inflammatory response. Clinical laboratory parameters and ECGs were also assessed. The follow-up period lasted 7 days. On Day 7, participants underwent clinical evaluations and completed a face-to-face structured interview. The secondary outcome was the characterization of the pharmacokinetics of the nasal formulations of artesunate and DHA in plasma.

### Pharmacokinetic analysis

A non-compartmental analysis (NCA) was used to derive individual pharmacokinetic parameters of artesunate and dihydroartemisinin, using PKanalix v.2024R1 (Simulations Plus, USA). Individual artesunate doses (mg) was converted to dihydroartemisinin equivalents, based on the relative difference in molecular weights (384.42 vs. 284.35 g/mol), for the analysis of dihydroartemisinin. All drug measurements were below the detection limit at 24 h after dose and therefore removed from the analysis. Of the remaining observations, the first drug measurement below the LLOQ in the terminal phase in an individual concentration-time profile was imputed with LLOQ/2 (i.e., 0.6 ng/mL for artesunate and 1.0 ng/mL for dihydroartemisinin), and all other LLOQ data was ignored. Total exposure up to the last measured concentration (AUC_last_) was calculated using the linear trapezoidal method for ascending concentrations and the logarithmic trapezoidal method for descending concentrations, as previously described^33^. The elimination constant (λ_Z_) was estimated by log-linear regression of a minimum of three observed concentration-time data in the terminal elimination phase. Terminal elimination half-life (t_1/2_) was calculated by (ln2/λ_Z_), and peak concentration (C_MAX_) and time to peak concentration (T_MAX_) were taken directly from the observed data. Total exposure (AUC_inf_) was calculated as AUC_last_ plus the extrapolated area in the terminal phase (C_last_ /λ_Z_). Total exposure was also normalised on individual dose (mg/kg), to assess dose-linearity. Pharmacokinetic parameters were summarised as median and range, and stratified by dose group.

### Investigational medicinal product stability testing

The IMP stability was tested with an accelerated stability assessment program (ASAP), every three months in the first year and every six months in the second year. Three primary batches of the IMP were prepared with the same artesunate powder and the same device batch as the trial batch. The preparation method and container closure system were identical, and all batches were produced concurrently with the trial batch. The overall quality of IMP batches placed on a formal stability study was representative of the quality of the material made on a production scale. Batches containing 20, 40, and 70 mg of artesunate powder loaded in nasal devices were stored in two climate chambers and analyzed by liquid chromatography-mass spectrometry. Climate temperatures and humidity were 25 °C ± 2 °C/60% relative humidity ± 5% and 40 °C ± 2 °C/75% relative humidity ± 5%. Artesunate analysis was performed using an Ultra-High Performance Liquid Chromatography system (Ultimate 3000, Thermo Fischer Scientific, Massachusetts, USA) coupled to a quadrupole-time-of-flight mass spectrometer (Impact II, Bruker, Massachusetts, USA).

### Qualitative evaluation of volunteers’ perception

To evaluate the influence of relevant attributes on participants’ perceptions after intranasal administration of artesunate, face-to-face structured interviews with a set of 17 questions were conducted one week post-administration^55^. The principal investigator informed each volunteer that the questionnaire was anonymous and would not be used for personal evaluation. The answers were collected using an electronic case report form. The attributes chosen for evaluation were derived through informal discussions with adults living in a malaria-endemic area and were collected on case report forms. The most important attribute identified was the potential for adverse events after nasal administration. Safety data that cannot be categorized and succinctly collected in predefined data fields were recorded in the comment section of the case report form.

### Statistical analysis

Sample size was chosen pragmatically, based on similar first-in-human dose-escalation studies^53^. The number of participants was deemed sufficient to generate meaningful safety and pharmacokinetic results. All participants were included in the safety and in pharmacokinetic populations. Descriptive statistics (mean, median, standard deviation, range, CV%) were used to report artesunate/DHA concentrations, calculated PK parameters, and adverse events.

## Supplementary Information

Below is the link to the electronic supplementary material.

Supplementary Material 1

## Data Availability

The datasets generated during and analysed during the current study are available from the corresponding author on reasonable request.

## Abbreviations

ART: Artesunate
DHA: Dihydroartemisinin
PK: Pharmacokinetic
IV: Intravenous
PO: Per os
IR: Intrarectal
IM: Intramuscular
C_max_: Maximal plasmatic concentration
T_max_: Time to maximum plasmatic concentration
t_1/2_: Half-life
AUC_last_: Area under the curve from time zero of the last quantifiable concentration
AUC_inf_: Area under the curve from time zero to infinite time
