# Supplementary material for: Safety, tolerability and pharmacokinetic properties of intranasal artesunate in a first in human phase 1 trial
Source: Sci Rep. 2026 Jul 6;16:31183. doi: 10.1038/s41598-026-56855-1 (PMC13642029; doi:10.1038/s41598-026-56855-1)
Supplement: Supplementary file 1 [file 41598_2026_56855_MOESM1_ESM.docx]

**Supplementary material**

**Safety, tolerability and pharmacokinetic properties of intranasal artesunate in a first in human phase 1 trial.**

Karim Traore^1,^*†, Anne-Lise Bienvenu^2,3,^*, Drissa Coulibaly^1^, Abdoulaye Kassoum Kone^1^, Amatigue Zeguime^1^, Karim Bengaly^1^, Mohamed Cisse^1^, Joel Tarning^4,5,^**, Mahamadou Ali Thera^1,^**, Stephane Picot^2,6,7,^**^,^ ª

**Inclusion Criteria**

Potential subjects are required to meet all of the following criteria for enrollment into the study:

1.	Healthy adults between the ages 18-25 (inclusive);

2.	Able and willing to provide written, informed consent;

3.	Able and willing to comply with all research requirements, in the opinion of the Investigator;

4.	Axillary temperature < 37.5°C

5.	Laboratory Criteria within 10 days before enrollment:

•	Hemoglobin ≥ 11.7 g/dL for women; ≥ 12.0 g/dL for men;

•	White Blood Cell count = 3,800-10,800 cells/mm3;

•	Platelets = >140,000/mm3;

**Exclusion criteria**

Subjects meeting any of the following criteria are not eligible for inclusion in this study.

1. Ages < 18 or > 25;
2. Pregnant or lactating female
3. Not able or not willing to provide written, informed consent;
4. Signs and symptoms of severe or non-severe malaria according to WHO 2015 criteria
5. Active infections including tuberculosis
6. Axillary temperature ≥ 37.5°C
7. Any confirmed or suspected immunosuppressive or immunodeficient condition, including human immunodeficiency virus (HIV) infection
8. Severe malnutrition (body mass index (BMI) less than 70% of median normalized WHO reference weight)
7. Severe vomiting (defined as more than 3 times in the 24 hours prior to inclusion in the study) or severe diarrhea (defined as more than 3 watery stools in the 24 hours prior to inclusion in the study)
8. Anemia (Hemoglobin level < 11.7 g/dL)
9. History of malignancy of any organ system (other than localized basal cell carcinoma of the skin or *in situ* cervical cancer), treated or untreated, within the past 5 years, regardless of whether there is evidence of local recurrence or metastases
10. Known chronic underlying disease such as sickle cell disease, and severe cardiac, renal, or hepatic impairment
11. Known active or uncontrolled ear, nose and throat (ENT) disease
12. Inability to tolerate oral medication (in tablet and/or liquid form)
13. Patients with prior antimalarial therapy within minimum of 4 weeks
14. Use of other investigational drugs within 5 half-lives of enrollment, or within 30 days or until the expected pharmacodynamic effect has returned to baseline, whichever is longer
15. History or family history of long QT syndrome or sudden cardiac death, or any other clinical condition known to prolong the QTc interval, such as history of symptomatic cardiac arrhythmias, clinically relevant bradycardia or severe heart disease
16. History of hypersensitivity to artesunate and dihydroartemisinin, or its excipients or to drugs of similar chemical classes
